# HIV-1 Adenoviral Vector Vaccines Expressing Multi-Trimeric BAFF and 4-1BBL Enhance T Cell Mediated Anti-Viral Immunity

**DOI:** 10.1371/journal.pone.0090100

**Published:** 2014-02-28

**Authors:** Saravana Kanagavelu, James M. Termini, Sachin Gupta, Francesca N. Raffa, Katherine A. Fuller, Yaelis Rivas, Sakhi Philip, Richard S. Kornbluth, Geoffrey W. Stone

**Affiliations:** 1 Department of Microbiology and Immunology, Miami Center for AIDS Research, and the Sylvester Comprehensive Cancer Center, University of Miami Miller School of Medicine, Miami, Florida, United States of America; 2 Multimeric Biotherapeutics, Inc., La Jolla, California, United States of America; 3 Department of Radiation Oncology, University of Miami Miller School of Medicine, Miami, Florida, United States of America; Deakin University, Australia

## Abstract

Adenoviral vectored vaccines have shown considerable promise but could be improved by molecular adjuvants. Ligands in the TNF superfamily (TNFSF) are potential adjuvants for adenoviral vector (Ad5) vaccines based on their central role in adaptive immunity. Many TNFSF ligands require aggregation beyond the trimeric state (multi-trimerization) for optimal biological function. Here we describe Ad5 vaccines for HIV-1 Gag antigen (Ad5-Gag) adjuvanted with the TNFSF ligands 4-1BBL, BAFF, GITRL and CD27L constructed as soluble multi-trimeric proteins via fusion to Surfactant Protein D (SP-D) as a multimerization scaffold. Mice were vaccinated with Ad5-Gag combined with Ad5 expressing one of the SP-D-TNFSF constructs or single-chain IL-12p70 as adjuvant. To evaluate vaccine-induced protection, mice were challenged with vaccinia virus expressing Gag (vaccinia-Gag) which is known to target the female genital tract, a major route of sexually acquired HIV-1 infection. In this system, SP-D-4-1BBL or SP-D-BAFF led to significantly reduced vaccinia-Gag replication when compared to Ad5-Gag alone. In contrast, IL-12p70, SP-D-CD27L and SP-D-GITRL were not protective. Histological examination following vaccinia-Gag challenge showed a dramatic lymphocytic infiltration into the uterus and ovaries of SP-D-4-1BBL and SP-D-BAFF-treated animals. By day 5 post challenge, proinflammatory cytokines in the tissue were reduced, consistent with the enhanced control over viral replication. Splenocytes had no specific immune markers that correlated with protection induced by SP-D-4-1BBL and SP-D-BAFF versus other groups. IL-12p70, despite lack of anti-viral efficacy, increased the total numbers of splenic dextramer positive CD8+ T cells, effector memory T cells, and effector Gag-specific CD8+ T cells, suggesting that these markers are poor predictors of anti-viral immunity in this model. In conclusion, soluble multi-trimeric 4-1BBL and BAFF adjuvants led to strong protection from vaccinia-Gag challenge, but the protection was independent of standard immune markers. Soluble multi-trimeric SP-D-4-1BBL and SP-D-BAFF provide a novel technology to enhance adenoviral vector vaccines against HIV-1.

## Introduction

The HIV pandemic continues to be a major concern worldwide and novel strategies are being investigated to develop effective HIV-1 prophylactic vaccines. Two complementary strategies are currently being pursued: (1) antibody-based vaccines to prevent initial infection, and (2) T cell-based vaccines to control HIV-1 replication in individuals with breakthrough viremia. Despite disappointing results from the Step clinical trial and related animal trials [1]–[5] adenoviral vectors continue to be evaluated as a component of HIV-1 vaccines, including prime/boost vaccine strategies [6]–[8]. Alternative serotypes, including Ad35 and Ad26, are also being investigated [9]–[16]. Despite encouraging results in animal models, a Phase II clinical trial of DNA prime/Ad5 boost vaccination was recently discontinued due to failure to protect against infection [17]. These data suggest that novel methods are required to alter the immune response generated by adenoviral vectors, potentially through the use of novel molecular adjuvants.

To date there have been a limited number of reports in the literature where adenoviral vector vaccines were enhanced with molecular adjuvants. For example, Ad5 expressing GM-CSF has been used in cancer immunotherapy to induce immune responses against irradiated tumor cells [18]. However, this approach is not directly applicable to infectious disease prophylactic vaccination. Similarly, adenovirus has been used for the delivery of GM-CSF or IL-12 to dendritic cells for DC vaccination strategies [19]. In contrast, DNA vaccine studies suggest that various immunostimulatory genes can improve T cell and antibody-mediated immunity [20]–[25]. For Ad5 vaccines, similar gene-based adjuvants are needed that enhance protection from viral challenge.

**Figure 1:**
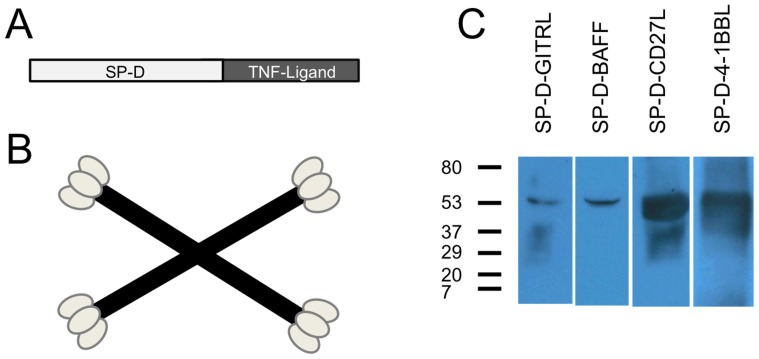
Construction of SP-D-TNFSFL Adenoviral vectors. A) Illustration of SP-D-TNFSFL cloning strategy. SP-D-TNFSFL genes were cloned by fusing the SP-D collagen-like domain to the extracellular domain of each ligand. Amino acids 1-256 of surfactant protein D were fused to the extracellular domain of each TNFSFL gene, including 4-1BBL, GITRL, BAFF, and CD27L. Details are provided in Materials and Methods B) Diagram of SP-D-TNFL multi-trimer structure. SP-D spontaneously forms four trimeric “arms” that are linked through disulfide bonding at the N-terminus to form 4-trimer structures [48], [49]. Shown here is a cartoon of the 4-trimer structure spontaneously formed by the collagen-like domain of SP-D followed by disulfide bonding of the SP-D N-terminus. C) Western blot of 293 cells infected with Ad5 constructs expressing SP-D-TNFL. 48 hours following viral vector transduction, supernatants were collected and run on a 4–15% Tris-Glycine SDS-PAGE gel in the presence of DTT before being probed with respective antibodies (see materials and methods). All viruses produced a 50–55 kDa recombinant protein expressing the expected TNF superfamily ligand.

TNF superfamily (TNFSF) ligands 4-1BBL, BAFF, GITRL, and CD70 (CD27L) play unique roles in the development of adaptive immunity and immunological memory and have been evaluated in a number of vaccine studies [26]–[29]. In work previously published by our group, we showed the importance of using soluble forms of TNFSF ligands that had many trimers and were capable of clustering their respective receptors. To this end, we prepared fusion proteins between surfactant protein-D (SP-D) and the extracellular domains of the TNFSF ligands (see Fig. 1B). The SP-D portion of the fusion protein contributes a self-assembling scaffold that holds four trimers of a TNFSF ligand. Using this strategy, it was shown that SP-D-CD40L, SP-D-CD27L, SP-D-4-1BBL, SP-D-RANKL, and SP-D-LIGHT stimulated T cell proliferation in vivo [30]–[32]. It was also observed that these SP-D-TNF superfamily ligands increased CD8+ T cell avidity, CD8/CD4 T cell proliferation 4 weeks post vaccination, as well as enhanced IL-2 secretion in memory T cell subsets [30].

The 4-1BB receptor (CD137) is expressed on the surface of T cells following T cell receptor (TCR) activation and is involved in the generation of T cell immunological memory through interaction with its cognate ligand 4-1BBL (CD137L) [33]. We have previously shown that DNA vaccines are enhanced with soluble multimeric 4-1BBL [30], and others have shown that protein vaccines can be enhanced by the use of agonistic antibodies to 4-1BB [34]. There is also evidence that full length 4-1BBL can enhance adenoviral vector vaccination [35].

Similar to 4-1BB, the receptors GITR and CD27 play complementary roles in the activation of T cells [36]–[40]. DNA vaccines encoding soluble multimers of GITRL [31], or peptide vaccines encoding anti-GITR agonistic antibody [41], can increase T cell responses. Vaccine studies have also been performed using antibodies to CD27 or its ligand CD27L (CD70). Soluble multimers of CD27L were able to enhance immune responses in mice vaccinated with Ova peptide [42]. More recently, we have shown that DNA vaccination with soluble multimers of CD27L can enhance T cell responses as measured by interferon gamma ELISPOT assay [30], though responses were not maintained long term.

The ligand BAFF is known to play a critical role in B cell memory and the development of long-lived plasma B cells [43]–[45]. Previous studies by our lab have highlighted the ability of soluble multimers of BAFF to enhance T cell immunity [30]. We propose that the activity of BAFF as a DNA vaccine adjuvant may be mediated by the expression of BAFF receptor (BAFF-R) on T cells.

In this report we investigated the ability of soluble multi-trimers of TNF superfamily ligands 4-1BBL, BAFF, GITRL and CD27L to enhance Ad5 viral vector vaccines. We show that Ad5 vectors encoding soluble multi-trimeric 4-1BBL or BAFF elicited protective immunity against vaccinia-Gag viral challenge. 4-1BBL and BAFF adjuvants also enhanced IFN-γ ELISPOT responses, and BAFF increased other markers of immunity, including the number of Gag-specific CD8+ T cells. In contrast, the molecular adjuvant IL-12p70 increased antigen-specific CD8+ T cell immune responses, yet failed to enhance protection from a vaccinia-Gag viral challenge. Surprisingly, BAFF and 4-1BBL-mediated protection was characterized by a dramatic inflammatory response in the reproductive tract of vaccinia-Gag challenged female mice, with high levels of lymphocyte infiltration. Despite this infiltration we observed low levels of pro-inflammatory cytokines, consistent with clearance of the virus. These data highlight a potentially unique mechanism of protection induced by multi-trimeric 4-1BBL and BAFF molecular adjuvants.

## Materials and Methods

### Construction of recombinant adenovirus 5 expressing Gag antigen or TNFSF-ligands

Replication defective adenovirus 5 (Ad5) was constructed expressing either codon-optimized Gag with a tissue plasminogen activator gene signal sequence [30] or murine versions of SP-D-CD27L, SP-D-4-1BBL, SP-D-GITRL, or SP-D-BAFF (detailed in [30], [31]), IL-12p70 formed as a single-chain combining the p35 and p40 subunits joined by a linker (Invivogen), or an irrelevant protein (green fluorescent protein, GFP). Briefly, constructs were originally cloned such that the gene contained mouse SP-D from the N-terminus to amino acid sequence ALFPDG. This was fused directly to each mouse TNFSFL extracellular domain, starting from the N-terminal amino acid sequences RTEPRP in 4-1BBL, SLKPTA in GITRL, LSKQQQ in CD27L, or AFQGPE in BAFF. Replication defective adenoviral constructs were produced as described by the manufacturer (AdEasy Adenoviral vector system, Agilent tech). Briefly, genes were PCR amplified and cloned into the pAdenoVator-CMV5 shuttle vector (Qbiogene). The constructs were sequenced to confirm correct gene expression. The CMV5-shuttle vectors were then electroporated into BJ5183 cells containing the pAdEasy-1 plasmid, allowing homologous recombination. The recombined pAdEasay-1 vector was isolated, linearized and transfected into AD293 cells (Stratagene). Following serial propagation in AD293 cells, the recombinant Ad5 virus was purified and concentrated using the Adeno-X Mega purification kit (Clontech). The concentration of viral particles (vp) was determined by measuring the absorbance at 260 nm and 280 nm, and calculated using the formula vp/ml = OD260 × viral dilution × 1.1×10ˆ12. To determine infectious units, viruses were titered using Adeno-x Rapid titer kit (Clontech).

### Western Blot analysis

1×10^6^ 293T cells were transduced with 1×10^6^ vp of each viral construct. After 48 hours, supernatant and cells were harvested for Western blot analysis. Cells were lysed in RIPA buffer (Biorad) and proteins were denatured with 2% SDS and reduced with 1% DTT before they were loaded into a 4–15% gradient Tris Glycene-SDS polyacrylamide gels (Bio-Rad), electrophoresed, and blotted onto PVDF membrane (Pierce). The membrane was blocked using 5% (w/v) dry milk and then probed with goat anti-mouse 4-1BBL or CD27L, or rat anti-mouse GITRL or BAFF (R&D Systems), followed by incubation with either anti-goat or anti-rat horseradish peroxidase-conjugated antibodies (Jackson Immunoresearch). The protein band was developed onto X-ray film using ECL detection reagent (Amersham).

### Mice and immunization schedule

Animals were housed at the University of Miami under the guidelines of the National Institutes of Health (NIH, Bethesda, MD). All animal experiments were performed in accordance with national and institutional guidance for animal care and were approved by the IACUC of the University of Miami. Female BALB/c mice (7–8 week old) were used for all experiments. Animals were immunized at a single timepoint or every two weeks. The number of vaccinations varied as noted. In all experiments mice were euthanized by CO_2_ asphyxiation. Two weeks following the final immunization, animals were sacrificed for T cell analyses. Mouse experiments were performed twice to confirm results, unless otherwise noted.

### Route of immunization

BALB/c mice (5 mice/group) were immunized with 1×10^9^ particles of Ad5-Gag virus per mouse either by intramuscular injection (100 ul) in the quadriceps muscle of both hind limbs, by i.p. injection (100 ul/mouse), or by tail vein injection (100 ul). For nasal immunization, mice were anesthetized and 50 ul of 1×10^9^ particles of virus was slowly delivered in one of the nostrils as previously described [46]. Mice were sacrificed 2 weeks following immunization and cellular and humoral responses were determined by ELISPOT and Gag specific IgG ELISA assay.

### Optimization of Ad5-Gag vaccine dose

Mice were vaccinated with increasing doses of Ad5-Gag (1×10^7^ to 1×10^10^ vp) by intramuscular injection into both hind quadriceps muscles. In a second experiment we compared combination vaccination with Ad5-Gag and Ad5 expressing either irrelevant protein (GFP) or Ad5-SP-D-CD27L. The optimal dose of Ad5-Gag was determined by Gag specific cellular and humoral responses two weeks following the immunization.

### Immunization Schedule

Based on route and dose optimization results, it was decided to evaluate adjuvants via i.m. vaccination. Ad5-Gag virus was combined with either Ad5-GFP or each Ad5-SP-D-TNFSF ligand adjuvant virus. Mice (5 per group) were injected with 1×10^6^ infectious units (IFU) of AD-5-Gag and 1×10^6^ IFU of Ad5-GFP, Ad5-IL-12 or Ad5-SP-D-TNFSF ligand adjuvant. 1×10^6^ IFU corresponded to 1×10^9^ vp for the optimization experiments. Ad5-GFP was used as a negative control while Ad5-IL-12 was used as a ‘gold standard’ for comparison of the relative activity of each SP-D-TNFSF ligand adjuvant.

### Splenocyte preparation

Two weeks following the final immunization, mice were euthanized and spleens removed. Single cell splenocyte preparations were obtained by passage through a 70 um nylon cell strainer (BD Falcon). Erythrocytes were depleted with ACK lysis buffer (Gibco) and splenocytes were washed thoroughly with R10 media (RPMI 1640 supplemented with 10% fetal bovine serum (FBS), 50 µM 2-mercaptomethanol, 100 U/ml of penicillin, 100 µg/ml streptomycin, and 10 mM HEPES).

### Enzyme linked immunospot (ELISPOT) assay

IFN-gamma, IL-2, and IL-4 ELISPOT assays were performed to determine antigen specific cytokine secretion from immunized mice splenocytes. ELISPOT assays were carried out per the manufacturer's protocol (R&D Systems) using 96-well MAIP plates (Millipore). Freshly prepared vaccinated mouse splenocytes (5×10^5^ cells/well) were added to each well of the plate, and stimulated for 18 h at 37°C, 5% CO2, in the presence of HIV-1 Gag peptide AMQMLKETI (5 ug/ml). For avidity ELISPOT assay, a range of peptide concentrations was used from 5 µg/ml to 10^−7^ µg/ml. A c-myc peptide (negative control) and PMA/Ionomycin (positive control) were also included to calculate the number of antigen-specific spots. After 18 h, spots were developed with AEC substrate kit (BD Bioscience), according to manufacturer's instructions. The membrane was read by automated reader (CTL Immunospot) for quantitative analyses of the number of IFN-gamma, IL-2 or IL-4 spots forming counts (SFC) per million cells plated, subtracting negative control values.

### Dextramer and Memory Staining

2×10^6^ PMBC were stained with PE H-2K^d^ AMQMLKETI MHC-I dextramer (Immudex) for 10 minutes in PBS with 5% FBS at room temperature. Anti-mouse CD3 Pac Blue, Anti-mouse CD8a PerCP, Anti-mouse CD127 APC (BD Bioscience) and Anti-mouse CD62L FITC (eBioscience) antibodies were added and incubated for 20 minutes at 4°C. Cells were washed twice with PBS 5% FBS then run immediately on a BD Biosciences LSR-Fortessa cell analyzer. CD3+CD8+ cells were analyzed for dextramer staining. The memory phenotype of dextramer positive CD8+ T cells was determined by analyzing the expression of CD62L and CD127 [47]. Central memory T cells were characterized as CD127+ CD62L+, effector memory CD127+ CD62L-, effector cells CD127- CD62L-, and transitional memory cells CD127- CD62L+.

### Intracellular cytokine staining

2×10^6^ splenocytes were stimulated with HIV-1 Gag peptide AMQMLKETI (5 ug/ml) for 6 hours at 37°C in the presence of 1 ul/ml GolgiPlug (BD Bioscience). Cells were washed twice with FACS Buffer (PBS, 1% BSA, 0.1% sodium azide, and 1 ul/ml GolgiPlug). Splenocytes were surface stained with anti-mouse CD3 Pac Blue, anti-mouse CD4 FITC, anti-mouse CD8a PerCP for 30 minutes before being fixed with 2% paraformaldehyde and permeabilized with 0.2% saponin. Intracellular cytokines were stained with anti-mouse IL-2 PE, anti-mouse IFN-γ PEcy7, and anti-mouse TNF-Alpha APC (BD Bioscience). After 3 washings, cells were fixed with 2% formalin and run immediately on a BD LSR Fortessa cell analyzer.

### [^3^H]-thymidine incorporation assay

Proliferative responses of T cells isolated from immunized mice two weeks post vaccination were determined using a standard [^3^H]-thymidine incorporation assay. 100 µl of spleen cell suspension (2×10^5^ cells/well) was plated in triplicate wells into 96-well round-bottom microtiter plate and stimulated using Gag protein (5 µg/ml), media control, or 10 µg/ml concanavalin A (positive control). The cells were incubated for 72 h before the addition of 1 µCi/well [^3^H]-thymidine. After 19 h, Cells were harvested onto fiberglass filters and radioactivity was measured in a liquid scintillation counter (Wallac Inc.). The results were calculated as cpm (mean ± SD of triplicate cultures).

### Elisa Assay for Anti-Gag IgG Responses

Anti-Gag antibody production was measured by ELISA assay. HIV-1 p55 Gag protein (10 µg/ml) was coated onto 96-well ELISA plates overnight at 4°C. Mice serum at different dilutions (1∶30, 1∶120, 1∶480 and 1∶1920) was added to the plates and incubated at room temperature for 2 h with shaking. Gag specific IgG antibodies were detected using alkaline phosphatase-conjugated goat anti-mouse IgG (Jackson Immunoresearch Inc.). Signal was developed using BluePhos substrate (KPL, Inc.). Plates were analyzed using a 96-well plate absorbance reader at 650 nm. Endpoint titers were calculated as the highest dilution that remained twice the baseline value.

### Flow Cytometry Analysis

All flow cytometric data was analyzed using FlowJo 7.6.4.

### Vaccinia Viral challenge

One month following vaccination, mice were challenged i.p. with 1×10^7^ vp vaccinia-Gag virus. Five days after challenge, mice were sacrificed and both ovaries and uterus were removed and homogenized in 500 ul PBS. For measurement of virus titers, samples were sonicated, and evaluated in triplicate by 10-fold serial dilution on CV-1 cells plated in 24 well plates. After 48 hour incubation, plates were stained with 0.1% (w/v) crystal violet in 20% ethanol. Plaques were counted to determine PFU of virus per total volume of ovary lysate.

### Ovary and Uterine Histology

Five days after vaccinia challenge, mice were sacrificed and both ovaries and uterus were removed and fixed in 10% formalin. Samples were imbedded in paraffin, sectioned, and stained with hematoxylin and eosin by the University of Miami Pathology Research Resources Histology Laboratory.

### Ovaria and Uterine Lysate Cytokine Bead Array (CBA) Analysis

Vaccinia challenge ovary lysates were analyzed using the Mouse Inflammatory Cytokine CBA kit (BD Bioscience) according to manufacturers instructions. Cytokine values were calculated and represented as pg/ml.

### Statistical analysis

Statistical analysis was performed using one-way Anova and Tukey's posthoc analysis. Individual comparisons between groups were performed using the Mann-Whitney test with Bonferroni adjustments to the alpha level. For all figures p values were labeled by asterisks for p<0.05 (*), p<0.01 (**), or p<0.001 (***). All statistical analysis was performed using GraphPad Prism 6 software.

### Ethics statement

All animal experiments were performed in accordance with national and institutional guidance for animal care and were approved by the IACUC of the University of Miami.

## Results

### Construction of adenoviral vectors containing TNF superfamily ligand adjuvants

Based on our previous studies with DNA vaccines adjuvants containing recombinant SP-D-TNF superfamily (TNFSF) ligands [30], we cloned constructs SP-D-BAFF, SP-D-CD27L, SP-D-GITRL and SP-D-4-1BBL into a replication-defective Ad5 viral vector system. Constructs were cloned such that mouse SP-D protein (from the N-terminus to amino acid sequence ALFPDG) was fused directly to the mouse TNFSF ligand extracellular domain (Fig. 1A). Based on biochemical analysis of full length SP-D and the SP-D-TNFSF ligand construct SP-D-CD40L [48]–[50], these SP-D-TNFSF ligand fusion constructs are expected to self-assemble into 12-mer structures as illustrated in Figure 1B. These fusion constructs were then cloned into an Ad5 shuttle vector containing the CMV-5 promoter [51] in order to increase protein expression levels. Construction of the final viral clone was confirmed by sequencing to ensure the proper nucleotide sequence for all recombinant gene constructs. Virus was purified from infected AD293 cells to produce a viral stock of each construct.

As shown in Fig. 1C, all Ad5-transduced 293 supernatants generated a band at a molecular weight of 50–55 kDa after probing with their respective anti-TNFSF ligand antibodies. These SP-D-TNFSF ligand constructs were previously shown to form a multimeric complex by western blots on a non-denaturing gel in the absence of SDS and DTT [30].

### Intramuscular injection of Ad5 gives superior CD8 T cell responses

**Figure 2:**
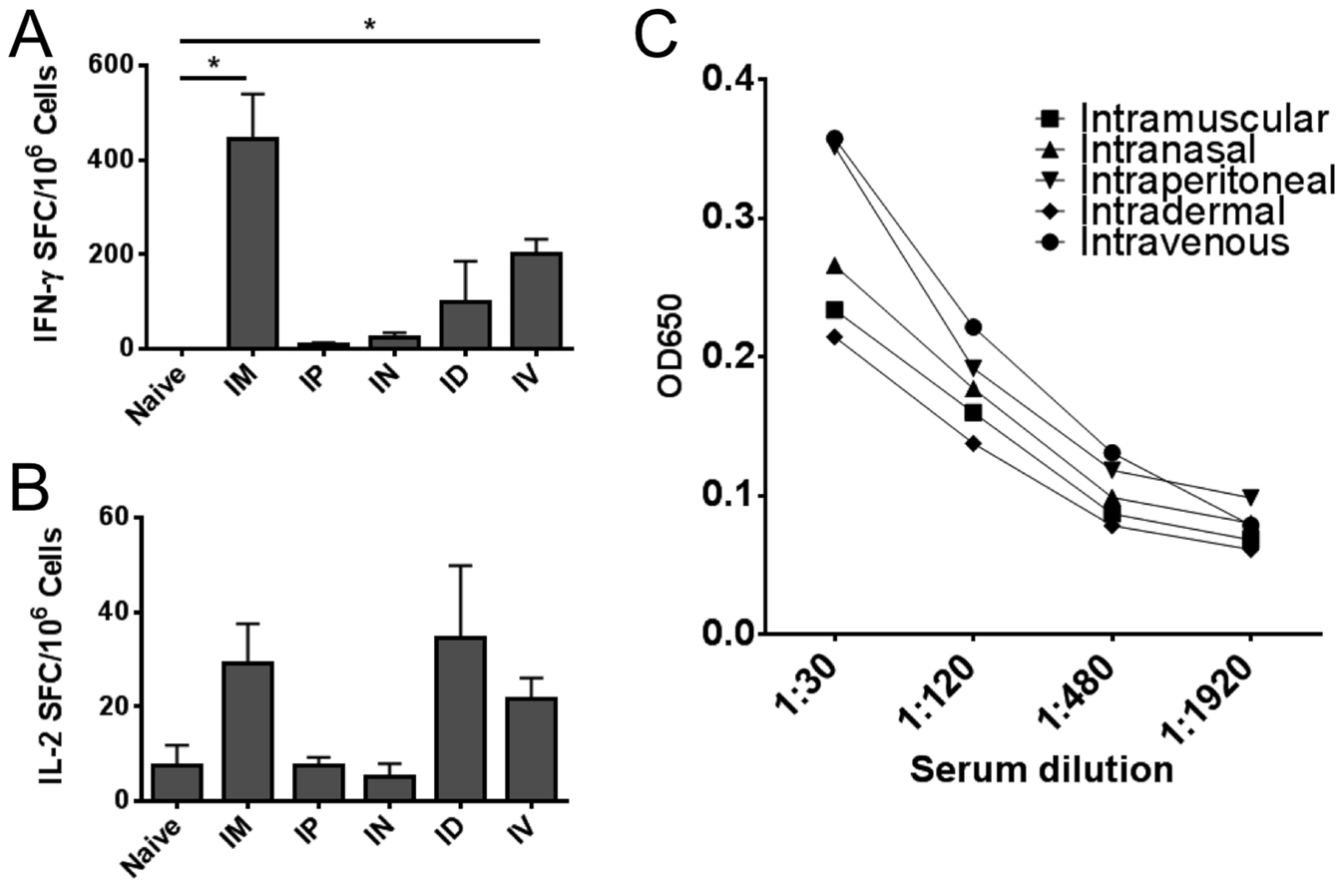
Intramuscular vaccination gives a superior immune response compared to other routes of injection. BALB/c mice were vaccinated once with 10^9^ viral particles of Ad5-Gag. Routes of injection included intramuscluar (i.m.), intraperitoneal (i.p.), intranasal (i.n.), intradermal (i.d.), or intravenous (i.v.). After 2 weeks, mice were sacrificed and spleens were removed for immune assay. Panels A and B: ELISPOT assays. A total of 2×10^5^ fresh splenocytes were cultured in a 96 well MAIP plate coated with IFN-γ (Panel A) or IL-2 capture antibody (Panel B), in the presence of 5 ug/ml HIV-1 Gag peptide AMQMLKETI. After 18 hours plates were developed with AEC substrate and counted in an automated plate reader. Data displayed as Spot-Forming Cells (SFC) per million splenocytes. Panel C: Anti-Gag IgG ELISA. Mice were bled prior to sacrifice and serum was isolated. Serum was cultured for 2 hours at indicated dilution on ELISA plates coated with HIV-1 p55 Gag protein (10 µg/ml). Plates were washed and probed with alkaline phosphatase-conjugated goat anti-mouse IgG antibody for 1 hour before developing with BluePhos substrate. Absorbance was measured at 650 nm.

Initially we evaluated the optimal route of Ad5 vaccination using an Ad5-Gag construct alone. Of all routes of vaccination tested, i.m. injection gave the highest IFN-γ ELISPOT responses (Fig. 2A). i.v. and i.d. injection were also effective at inducing IFN-γ ELISPOT responses but not to the level of i.m. injection. Enhanced IL-2 ELISPOT responses were observed for i.m. i.d. and i.v. routes of vaccination, but did not reach statistical significance (Fig. 2B). Anti-Gag antibody responses were weak following Ad5 vaccination. All routes of vaccination induced modest levels of IgG antibody titers against Gag. ELISA responses were above background in the 1∶480 dilution range but not at 1∶1920 (Fig. 2C). No particular route of injection gave significantly higher IgG responses compared to any other route of injection.

### 10^9^ Viral Particles is the optimal dose for vaccination

**Figure 3:**
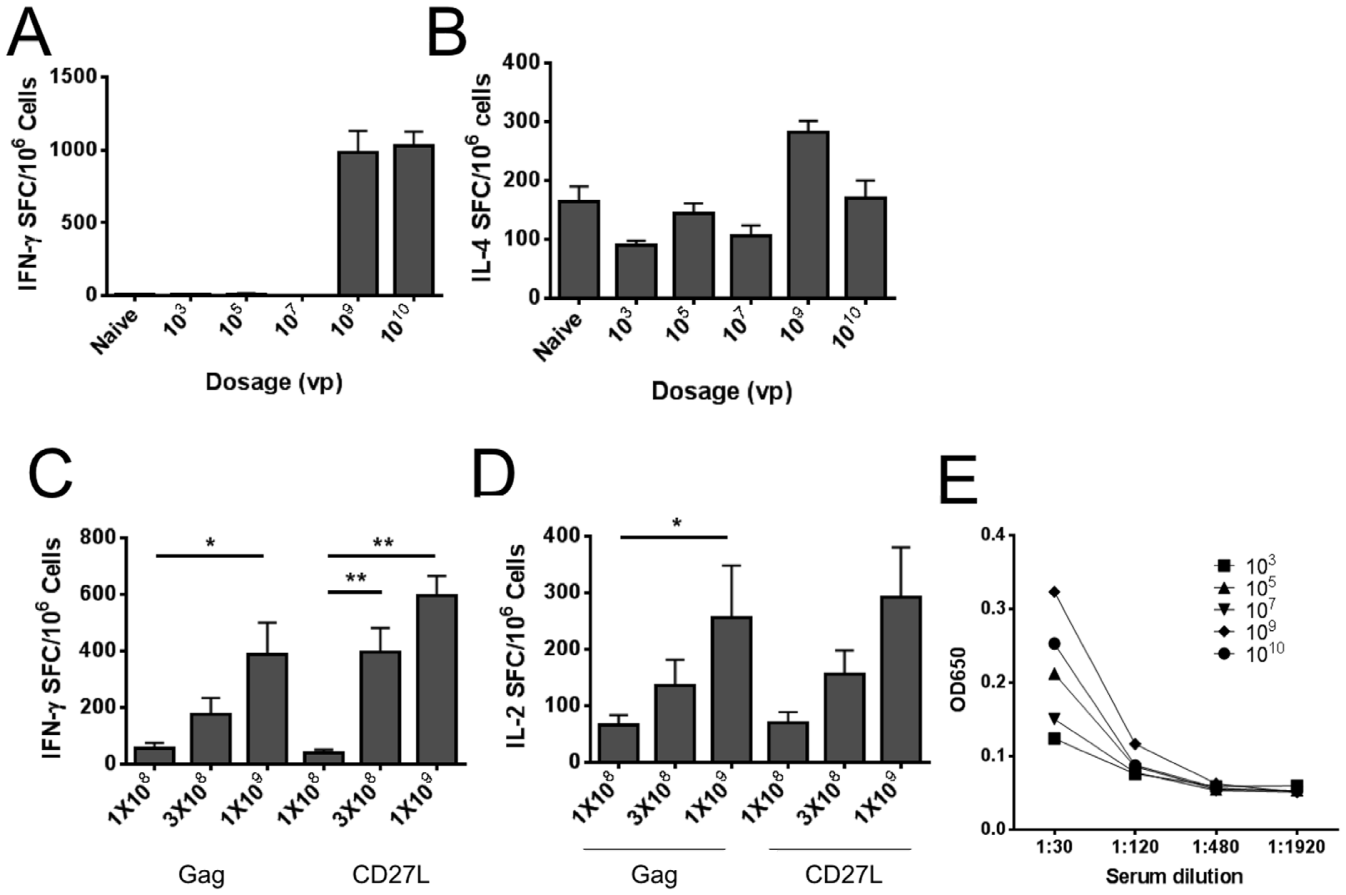
Optimal dosage for adjuvanted vaccines. BALB/c mice (5 per group) were vaccinated once with the indicated dose of Ad5-Gag. Panel D and Panel E: Equal concentrations of AD5-Gag & SP-D-CD27L or irrelevant protein (GFP) virus were added where specified. 2×10^5^ fresh splenocytes were cultured in a 96 well MAIP plate coated with IFN-γ (Panel A and Panel C), IL-4 (Panel B), or IL-2 capture antibody (Panel D), in the presence of 5 ug/ml HIV-1 Gag peptide AMQMLKETI. After 18 hours plates were developed with AEC substrate and counted in an automated plate reader. Data displayed as Spot-Forming Cells (SFC) per million splenocytes. Panel C: Anti-Gag IgG ELISA. Before sacrifice, mice were bled and serum was isolated. Serum at indicated dilution was cultured for 2 hours on ELISA plates coated with HIV-1 p55 Gag protein (10 µg/ml). Plates were washed and probed with alkaline phosphatase-conjugated goat anti-mouse IgG antibody for 1 hour before developing with BluePhos substrate. Absorbance was measured at 650 nm.

Based upon route-of-vaccination results, it was decided to determine the optimal dose to observe an adjuvant effect using i.m. injection. We compared a range of Ad5 vaccination doses from 10^3^ to 10^10^ viral particles (vp) per injection. Interestingly, no IFN-γ responses were observed at doses of 10^3^, 10^5^, and 10^7^ vp/mouse while 10^9^ vp induced a measureable IFN-γ response by ELISPOT (Fig. 3A). Increasing the dose to 10^10^ vp did not generate a significant increase in IFN-γ SFC compared to 10^9^ vp. IL-4 responses were moderate in all groups, with a trend to higher IL-4 ELISPOT responses at 10^9^ and 10^10^ vp per injection (Fig. 3B). Consistent with Figure 2C, antibody responses were minimal following i.m. injection. The highest antibody titers were observed at 10^9^ vp, however antibody titers were at background levels for 1∶480 or 1∶1920 dilutions (Fig. 3E).

To evaluate the optimal dose to measure adjuvant responses, we tested increasing doses of Ad5-Gag + Ad5-GFP (irrelevant protein) and compared this to Ad5-Gag + Ad5-SP-D-CD27L adjuvant. Consistent with Fig3A, increasing the Ad5 vaccine dose led to significant increases in both IFN-γ and IL-2 responses for control and adjuvant groups (Fig. 3C and 3D). We observed a trend toward higher IFN-γ ELISPOT responses comparing SP-D-CD27L adjuvant to GFP control at 3×10^8^ and 1×10^9^ vp doses (p = 0.07 and p = 0.15 respectively), but this did not reach statistical significance.

### Vaccination with Ad5-Gag in Combination with SP-D-TNFSF Ligands Can Enhance Protection in a vaccinia challenge model

**Figure 4:**
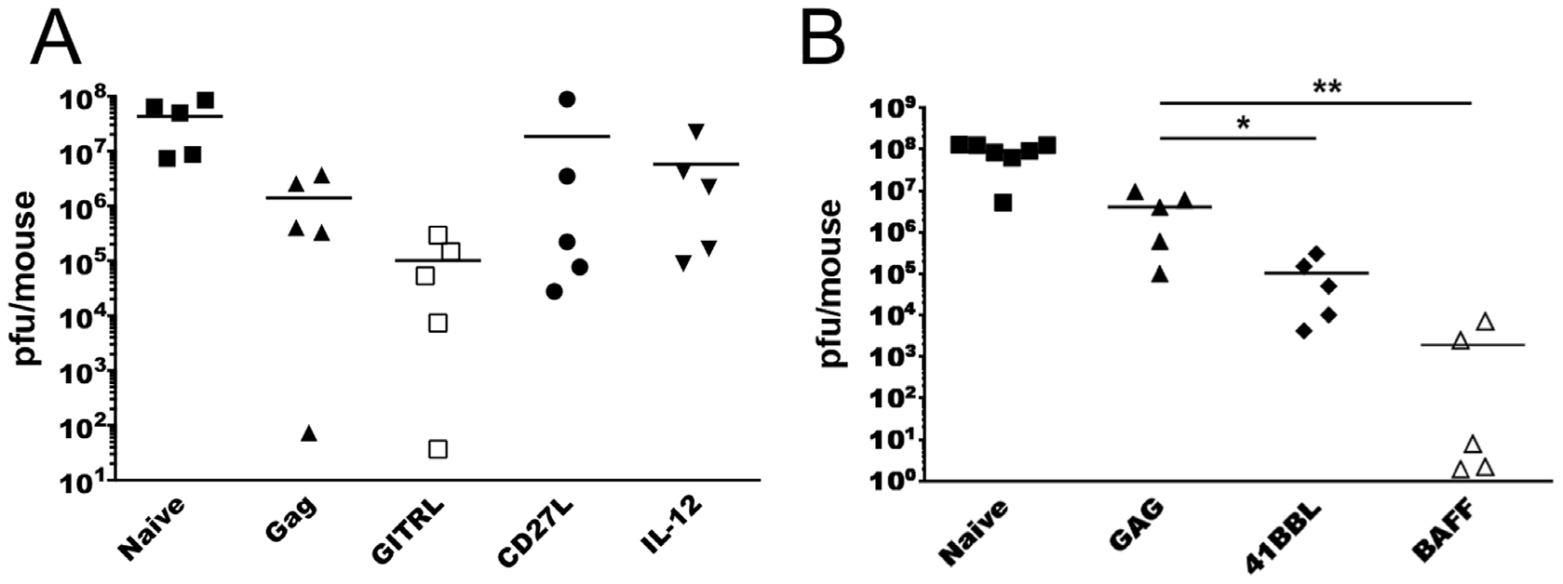
Vaccination with Ad5-SP-D-4-1BBL or Ad5-SP-D-BAFF adjuvants enhance protection from a vaccinia-Gag viral challenge. Panel A: BALB/c mice (5 per group) were immunized with Ad5-Gag with or without Ad5-SP-D-TNFSF ligand adjuvant constructs. Mice were vaccinated twice, two weeks apart. One month following the final vaccination, mice were challenged IP with 10^7^ VP vaccinia-Gag. Five days following vaccinia challenge mice were sacrificed and ovaries and uterus were removed, homogenized, and vaccinia virus was titered on Vero cells at various dilutions. After staining with crystal violet, viral plaques were counted and plaque-forming units (PFU) per mouse were calculated based on the total volume of ovary and uterus lysate. Panel B: Experiment similar to Panel A using SP-D-4-1BBL and SP-D-BAFF as vaccine adjuvants.

Next we evaluated our four SP-D-TNFSFL adjuvant constructs in a vaccinia-Gag challenge assay. In an initial experiment testing Ad5-SP-D-GITRL, Ad5-SP-D-CD27L and the gold standard adjuvant IL-12p70 as adjuvants, no group showed a significant reduction in tissue viral load compared to Ad5-Gag + Ad5-GFP (Fig 4A). The adjuvant Ad5-IL-12p70 was unable to reduce viral loads compared to Ad5-Gag + Ad5-GFP. The SP-D-GITRL adjuvant reduced mean viral load compared to other groups. However, this did not reach statistical significance at 5 mice per group. A higher n value will likely be required for the SP-D-GITRL group to properly determine statistical differences compared to antigen alone.

Next we evaluated SP-D-4-1BBL and SP-D-BAFF. Both adjuvants significantly reduced vaccinia titers compared to Ad5-Gag + Ad5-GFP (Fig 4B). Importantly, the addition of Ad5-SP-D-BAFF adjuvant decreased vaccinia-Gag titers below the limit of detection in 3 out of 5 animals. In a repeat experiment SP-D-4-1BBL, SP-D-BAFF and SP-D-GITRL significantly (p<0.05) reduced mean viral pfu/mouse compared to Ad5-Gag alone (data not shown), confirming the reproducibility of these results.

### Vaccination with SP-D-4-1BBL or SP-D-BAFF adjuvants leads to distinct inflammatory and histological profiles

**Figure 5:**
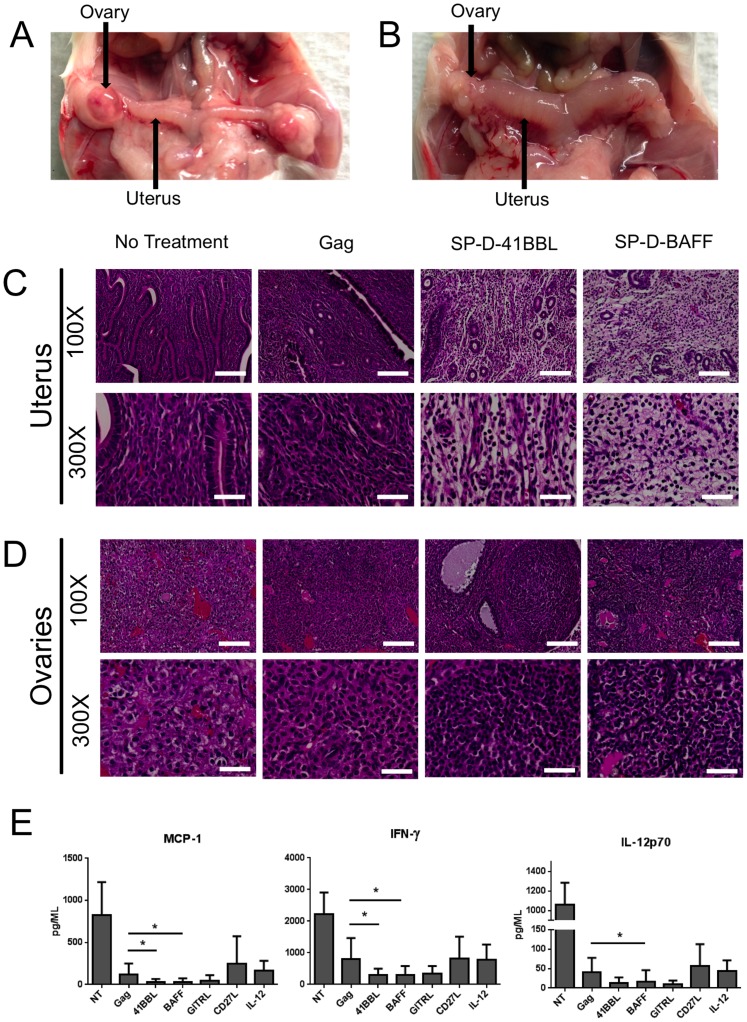
SP-D-4-1BBL and SP-D-BAFF induce distinct inflammatory responses and lymphocytic infiltration in the uterus and ovaries of vaccinia-Gag challenged animals. BALB/c mice (5 per group) were immunized with Ad5-Gag with or without Ad5-TNFSF ligand adjuvant twice, two weeks apart. One month following the final vaccination, mice were challenged i.p. with 10^7^ vp vaccinia-Gag virus. Five days following challenge, mice were sacrificed and ovaries and uterus were dissected. Panel A: Uterus and ovaries in unvaccinated mouse challenged with vaccinia-Gag. Panel B: Uterus and ovaries in mouse vaccinated with Ad5-Gag plus Ad5-SP-D-4-1BBL. Note the enlargement and dusky erythema in the uterine cornu when AD5-SP-D-4-1BBL was used as the adjuvant. Panels C and D: Histology of unvaccinated and vaccinated mice challenged with vaccinia-Gag studies after formalin fixation, paraffin embedding, and staining with hematoxylin and eosin. Samples were examined at 100× and 300× magnification. White scale bar represents 200 µm (100×) or 40 µm (300×). Panel C shows the uterus histology following virus challenge of unvaccinated mice, Ad5-Gag vaccinated mice, Ad5-Gag/Ad5-SP-D-4-1BBL vaccinated mice, and Ad5-Gag/Ad5-SP-D-BAFF vaccinated mice. Panel D shows the ovaries of the same groups of mice as Panel C. Panel E: Ovary and uterus lysates were analyzed for cytokines using the Mouse Inflammatory Cytokine CBA Kit (BD Bioscience). Data pooled from experiments shown in Panels C and D.

It became apparent upon dissection that SP-D-4-1BBL or SP-D-BAFF groups induced a strong inflammatory response in the uterus and ovaries of these animals at 5 days post vaccinia-Gag challenge. Animals vaccinated with Ad5-Gag + Ad5-SP-D-4-1BBL or Ad5-SP-D-BAFF displayed normal size ovaries but a markedly enlarged uterus and reproductive tract (Fig. 5B and data not shown). In contrast, unvaccinated animals or animals receiving other adjuvants (IL-12p70, CD27L, GITRL) did not show an enlarged uterus (Figure 5A and data not shown). Mice with high viral titers displayed enlarged ovaries surrounded by a fluid filled sac, typical to vaccinia infection (Figure 5A). To further explore this inflammatory response, animals were vaccinated and then challenged with vaccinia-Gag as before. Ovaries and uterus were dissected, embedded in paraffin, and H&E staining was performed. The uteruses of mice vaccinated with SP-D-4-1BBL or SP-D-BAFF adjuvants displayed a distinct morphology compared to other groups (Fig 5C). Unvaccinated and Gag-only vaccinated animals displayed normal uterus morphology with healthy tissue and no gaps between cells (Fig 5C). In contrast, uterus from animals vaccinated with SP-D-4-1BBL or SP-D-BAFF adjuvants showed general edema, vasculitis, and high levels of lymphocyte infiltration. Ovaries were also examined. Vaccination with SP-D-4-1BBL or SP-D-BAFF adjuvants led to increased lymphocyte infiltration into the ovaries and reduced tissue destruction compared to both unvaccinated and Gag vaccinated animals (Fig 5D).

### Vaccination with SP-D-4-1BBL and SP-D-BAFF adjuvants decrease proinflammatory cytokines in response to vaccinia challenge

Based on the high level of T cell infiltration and edema we observed in the uterus of animals vaccinated with SP-D-4-1BBL and SP-D-BAFF, we examined cytokine levels in the ovary and reproductive tract at 5-days post challenge. Ovary/uterus lysates from vaccinia challenge experiments were analyzed by cytometric bead array for proinflammatory cytokines MCP-1, IFN-γ, and IL-12p70. Interestingly, at 5 days post vaccinia challenge, SP-D-4-1BBL- and SP-D-BAFF-vaccinated animals showed reduced levels of MCP-1, IFN- γ, and IL-12p70 cytokines compared to unvaccinated animals or animals vaccinated with Ad5-Gag alone. We did not observe difference between groups for cytokines IL-10, TNF-α, and IL-6 (data not shown). Both SP-D-CD27L and IL-12 adjuvants showed similar cytokine profiles to that of Ad5-Gag alone. SP-D-GITRL showed a trend toward decreased proinflammatory cytokine levels (Fig. 5E).

### SP-D-4-1BBL and SP-D-BAFF adjuvants increase markers of immune activation

**Figure 6:**
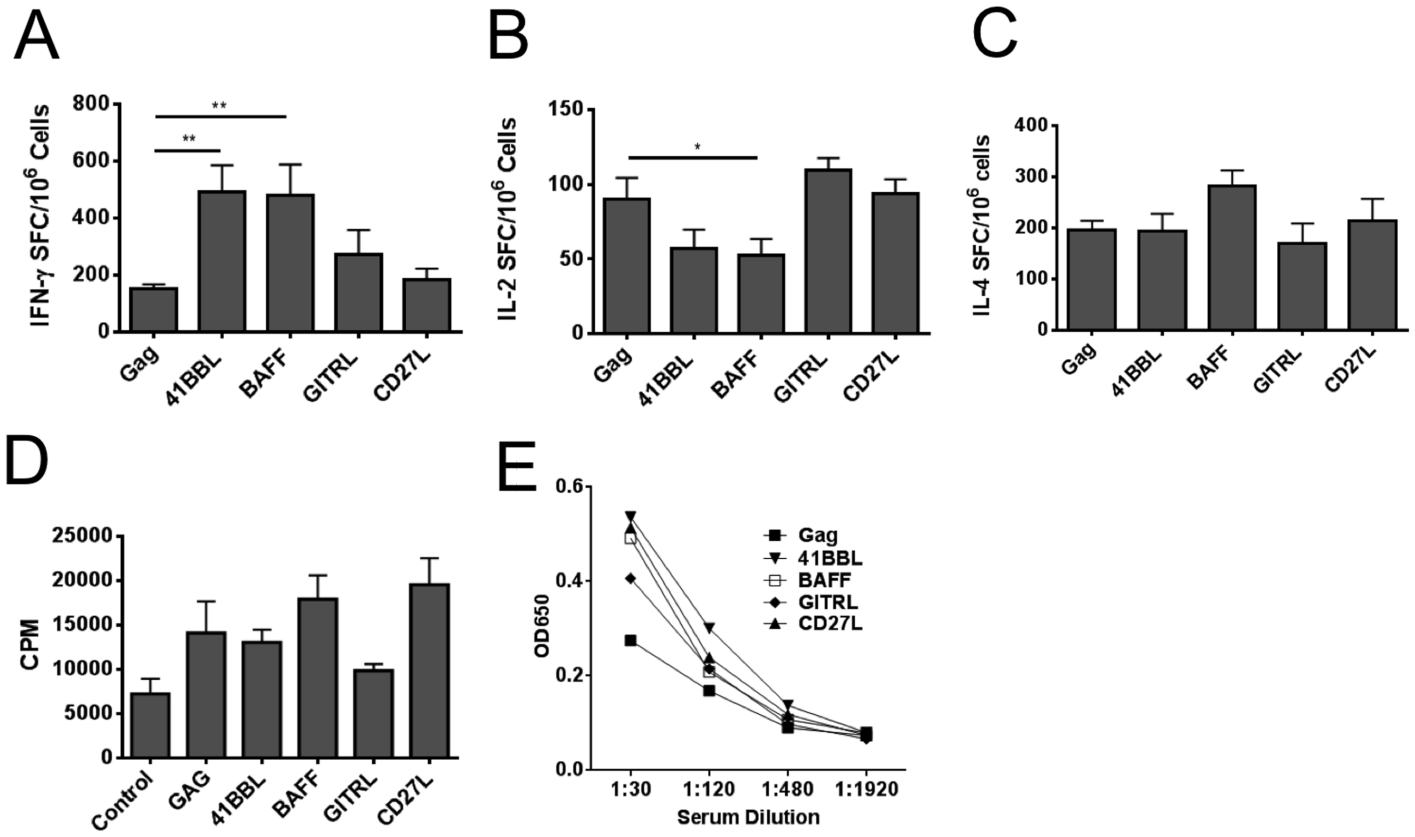
Vaccination with SP-D-4-1BBL and SP-D-BAFF adjuvants increase IFN-γ antigen-specific immune responses. BALB/c mice (5 per group) were vaccinated i.m. with 10^9^ viral particles of Ad5-Gag plus 10^9^ viral particles of either Ad5-SP-D-TNFSF ligand or Ad5-GFP control. Mice were vaccinated twice at 2-week intervals. Two weeks following the final vaccination, mice were sacrificed and spleens removed for immune assays. Then 2×10^5^ fresh splenocytes were cultured in a 96 well MAIP plate coated with Panel A: anti-IFN-γ, Panel B: anti-IL-2, or Panel C: anti-IL-4 capture antibody in the presence of 5 ug/ml HIV-1 Gag peptide AMQMLKETI. After 18 hours, plates were washed, incubated with secondary antibody and developed with AEC substrate. Plates were counted in an automated plate reader. Data are shown as Spot-Forming Cells (SFC) per million splenocytes. Panel D: [^3^H]-thymidine incorporation proliferation assay. 2×10^5^ cells were plated in triplicate wells into 96-well plate and stimulated using Gag p55 protein (5 µg/ml) for 3 days. 1 µCi/well [^3^H]-thymidine was added and cultured for an additional 19 h. Cells were harvested and radioactivity was measured by scintillation counter. Proliferation is displayed as counts per minute (CPM). E) Anti-Gag IgG Elisa. Mice were bled prior to sacrifice and serum isolated. Serum, at indicated dilutions, was cultured for 2 hours on ELISA plates coated with HIV-1 p55 Gag protein (10 µg/ml). Plates were washed and probed with alkaline phosphatase-conjugated goat anti-mouse IgG antibody for 1 hour before developing with BluePhos substrate. Absorbance was measured at 650 nm.

Next, the immune responses to Ad5-Gag vaccines containing the various TNFSF ligand constructs were examined to determine if any immune assays correlated with enhanced viral protection. Both Ad5-SP-D-4-1BBL and Ad5-SP-D-BAFF adjuvants significantly increased IFN-γ SFC by ELISPOT assay when compared to Ad5-Gag + Ad5-GFP (Fig. 6A). Adjuvants SP-D-GITRL and SP-D-CD27L did not significantly increase IFN-γ SFC compared to Ad5-Gag alone, however SP-D-GITRL did show a trend to lower viral load and requires future studies with a larger number of animals. In terms of IL-2 responses, there was a significant decrease in IL-2 SFC for the SP-D-BAFF vaccine group (Fig. 6B). All vaccines gave similar IL-4 ELISPOT responses, and we observed no significant differences between groups (Fig. 6C). Proliferation in response to culture with Gag protein was also analyzed. Compared to Ad5-Gag + Ad5-GFP, both SP-D-BAFF and SP-D-CD27L adjuvants moderately increased proliferation as measured by ^3^H incorporation (Fig. 6D), but this response did not reach statistical significance. IgG antibody titers were also analyzed. As shown in Figure 3C, Ad5 vaccination elicited only minimal antibody responses to Gag. There was a trend in all adjuvant groups toward increased IgG responses compared to Gag + GFP, but values did not reach statistical significance (Fig. 6E). These assays were repeated in a second experiment, again showing a significant (p<0.05) increase in IFN-γ ELISPOT responses for SP-D-4-1BBL and SP-D-BAFF (data not shown).

### SP-D-BAFF and SP-D-4-1BBL adjuvants induce unique CD8+ memory T cell phenotypes

**Figure 7:**
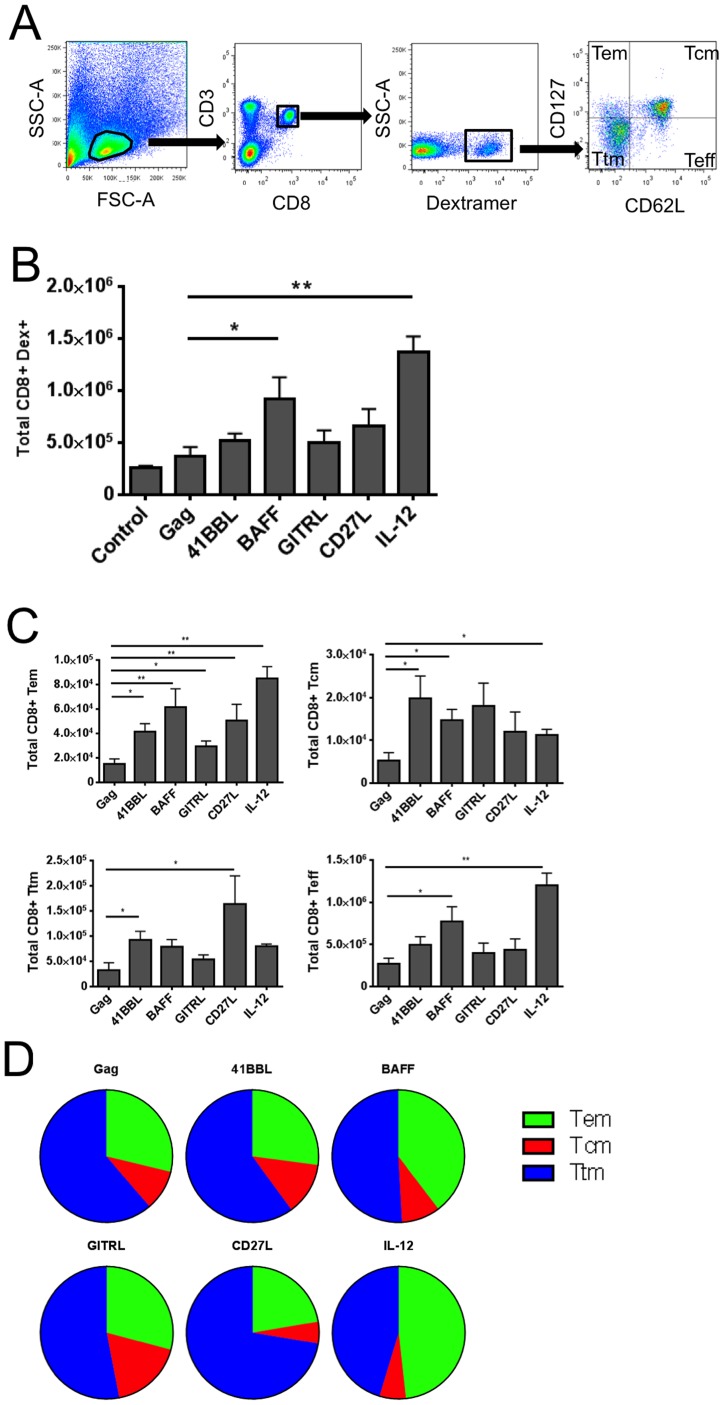
SP-D-4-1BBL, SP-D-BAFF, and IL-12p70 induce distinct memory T cell phenotypes. 2×10^6^ Fresh splenocytes were stained with PE-conjugated H-2K^d^ AMQMLKETI MHC-I Dextramer along with CD3, CD8, CD127, and CD62L antibodies for 30 minutes before being washed and analyzed by flow cytometry. Panel A: Diagram of flow cytometry gating strategy. Panel B: Absolute numbers of CD8+ Dex+ T cells per spleen. Panel C: Absolute numbers of CD8+ Dex+ T cell memory phenotypes per spleen. T_cm_ central memory, T_em_ effector memory, T_tm_ transitional memory, T_eff_ terminally differentiated effector. Panel D: Pie chart comparison of the relative numbers of each memory phenotype. The total number of each phenotype was graphed as a proportion of the total number of memory CD8+ DEX+ T cells per spleen.

Next we evaluated Gag peptide/MHC-I dextramer binding and the memory phenotypes of Gag-specific cells to determine whether any immune responses correlated with enhanced protection during vaccinia-Gag challenge. To evaluate memory phenotypes, lymphocytes were gated using a forward scatter gate for lymphocyte-sized cells, followed by gating on CD3 and CD8 markers. CD3+ CD8+ T cells were analyzed for Gag peptide AMQMLKETI-specific dextramer staining. Dextramer positive (Dex+) cells were then analyzed for their expression of CD62L and CD127 to characterize memory phenotypes (Fig. 7A). Ad5-Gag + Ad5-GFP vaccination induced only modest Gag-specific response compared to Ad5 expressing an irrelevant antigen (gp100) (Fig. 7B). SP-D-BAFF and IL-12p70 adjuvants both induced a significant increase in Gag specific dextramer positive CD8 T cells compared to Gag + GFP. Other adjuvants tested increased the number of CD3+CD8+Dex+ cells, but these did not reach statistical significance compared to Ad5-Gag + Ad5-GFP. We next analyzed the Dex+ population for total numbers of Dex+ CD8+ T cells of particular memory phenotypes (Fig. 7C). SP-D-BAFF, SP-D-4-1BBL, and IL-12p70 significantly increased the total number of Dex+ central memory CD8+ T cells compared to Ad5-Gag + Ad5-GFP. All adjuvants significantly increased the total number of Dex+ effector memory cells compared to Ad5-Gag + Ad5-GFP. Comparing the relative proportion of each phenotype, IL-12p70 induced more of an effector memory and terminally differentiated effector phenotype while SP-D-4-1BBL, SP-D-BAFF, and SP-D-GITRL induced more of a central memory and transitional memory phenotype (Fig. 7D).

### TNF-α Intracellular Staining and Immune correlation

**Figure 8:**
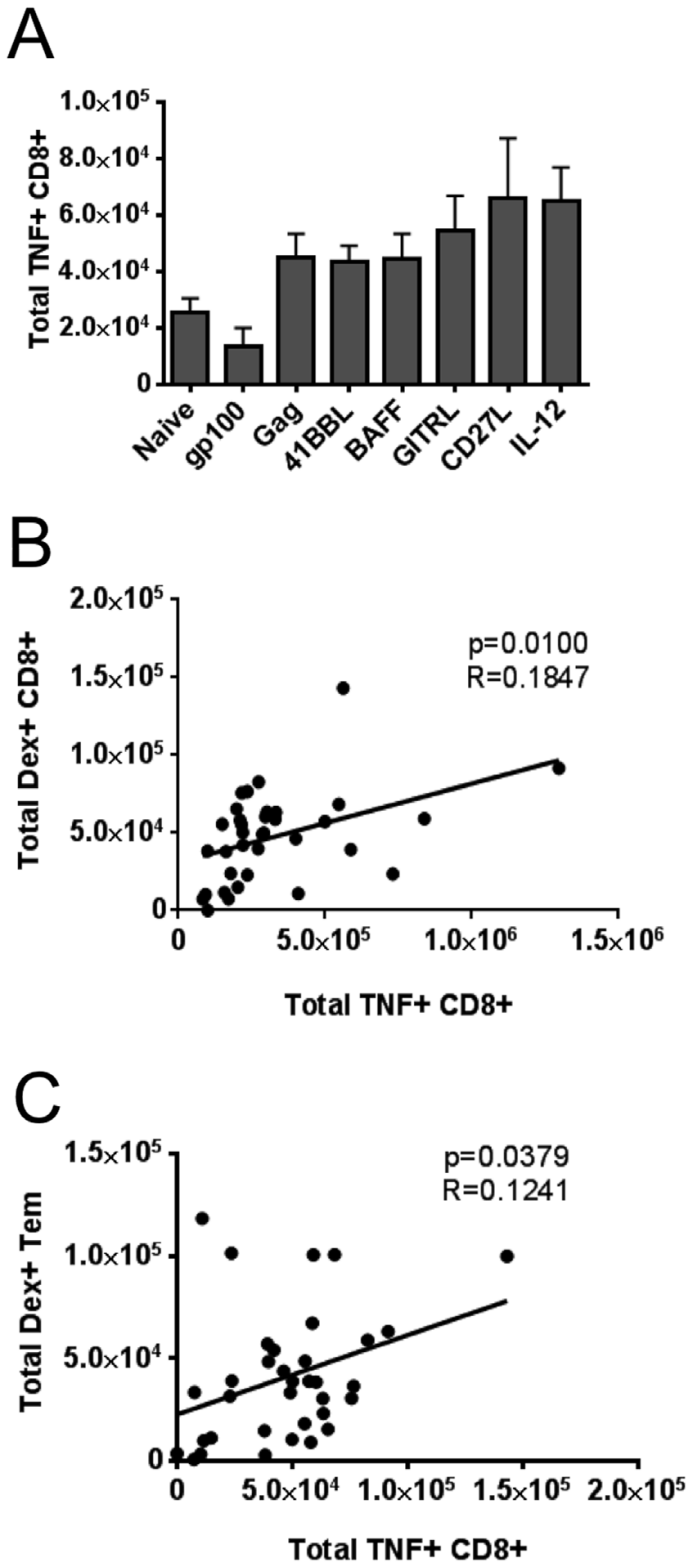
Intracellular cytokine staining and correlation between immune responses. Panel A: Total number of TNF-producing CD8+ T cells. 2×10^6^ splenocytes were stimulated with HIV-1 Gag peptide AMQMLKETI (5 ug/ml) for 6 hours in the presence of 1 ul/ml GolgiPlug to promote retention of TNF inside cells. Cells were washed twice then surface stained with anti-mouse CD3, CD4, and CD8α for 30 minutes before being fixed and permeabilized. Cells were then intracellularly stained with anti-mouse TNF-α antibody. After wash and fixation, cells were run immediately on an LSR-Fortessa flow cytometer (BD Biosciences). Absolute numbers of TNF+ CD8+ T cells were determined per spleen. Panel B: The number of cells expressing TNF after stimulation with Gag peptide was significantly correlated with the overall number of Gag-specific, dexamer-positive CD8+ T cells. Panel C: The number of cells expressing TNF after stimulation with Gag peptide was significantly correlated with the number of Gag-specific, dexamer-positive T effector memory (Tem) CD8+ T cells defined as cells that were CD3+ CD8+ CD127+ CD62L-. Statistical values for correlation were calculated using GraphPad Prism 6 software.

We also evaluated intracellular staining for TNF-α as a marker of immune responses. We observed no significant difference in the absolute numbers of TNF+ CD8+ T cells following vaccination with TNFSF ligand adjuvants when compared to Ad5-Gag + Ad5-GFP (Fig. 8A). There was trend to higher numbers of CD8+ TNF+ cells with both SP-D-CD27L and IL-12p70 adjuvants (p = 0.66 and p = 0.15 respectively) compared to control. However, both SP-D-4-1BBL and SP-D-BAFF displayed comparable levels of CD8+ TNF+ cells compared to animals vaccinated with Ad5-Gag + Ad5-GFP. We then performed correlation analysis for each mouse. TNF production positively correlated with the amount of Dex+ CD8+ T cells present (p = 0.010, r^2^ = 0.1847) (Fig. 8B). TNF production also correlated with the total number of Dex+ CD8+ effector memory T cells (p = 0.0379, r^2^ = 0.1241) (Fig. 8C).

## Discussion

These studies evaluated whether Ad5 HIV-1 vaccines could be enhanced with TNF superfamily ligand derived adjuvants. We evaluated adjuvants composed of multi-trimeric recombinant versions of 4-1BBL, BAFF, GITRL, and CD27L. Currently there are a limited number of studies where Ad5 vector vaccines have been enhanced with molecular adjuvants, including the use of full-length 4-1BBL and heat-labile enterotoxin [35], [52]. Other studies have evaluated Ad5-based adjuvants to enhance cell-based immunotherapies [18], [19]. None of these studies have shown enhancement of Ad5 vaccine mediated T cell immune responses with conventional adjuvants such as GM-CSF, highlighting the importance of developing novel adjuvants to increase T cell mediated immunity from Ad5 viral vector vaccines.

Following construction of Ad5 viral vectors expressing SP-D-GITRL, SP-D-BAFF, SP-D-CD27L and SP-D-4-1BBL we observed a distinct band by Western blot at the expected molecular weight of 50–55 kDa. Differences were observed in the intensity of each band, however these differences are likely to reflect variation in the detection antibodies used for each construct. Antibodies were not available against the SP-D collagen-like domain, and therefore we cannot directly compare expression levels for all constructs. Nonetheless, ELISA assays were performed on supernatant from transduced 293 cells, and similar concentrations of each fusion were observed when compared to a known quantity of recombinant TNFSFL protein (data not shown).

Initially we tested the optimal route of Ad5 vaccine delivery. Intramuscular injection gave the highest IFN-γ ELISPOT responses in our animal model. The i.m. route also generated a trend toward higher IL-2 ELISPOT levels. Based on these data we used i.m. injection as our primary route of delivery for this project. However, we also observed high IL-2 CD8+ T cell responses by both i.d. and i.v. injection, suggesting future studies to evaluate whether TNF superfamily ligands can adjuvant i.d. and i.v. routes of injection for Ad5 vaccines.

Based on dosage experiments (Fig. 3), 10^9^ viral particles (vp) was the minimum effective dosage for vaccination. No increase in IFN-γ or IL-2 ELISPOT responses was observed at the higher dose of 10^10^ vp. Although Gag responses could not be increased at dosages above 10^9^ vp, we evaluated whether immune responses could be increased by the use of SP-D-CD27L adjuvant. When Ad5-SP-D-CD27L was combined with the Ad5-Gag, there was a trend to increased Gag specific IFN-γ and IL-2 responses compared to when Ad5-GFP control virus was used. These data suggest that 10^9^ vp of Ad5-Gag and 10^9^ vp of Ad5-TNFSF ligand is the optimal dose for vaccination studies. We also observed an increase in IL-2 ELISPOT responses at increasing dosage. Previous reports have shown that IL-2 secreting CD8+ T cells can proliferate in a CD4-independent manner [53]. In addition, evaluation of polyfunctional T cell responses suggests that the size of the IL-2 secreting T cell population correlates with the overall polyfunctional T cell response and viral control [11], [54]. Increased IL-2 production as measured by ELISPOT has also been shown to correlate with an increase in memory responses [11], [54]. Given that i.m., i.d., and i.v. injection showed increased IL-2 ELISPOT responses, these data support a model whereby these routes of immunization may enhance memory T cell development. This enhanced memory response would be expected to lead to enhanced T cell mediated protection from viral infection, a key goal in HIV vaccine research.

We observed only a modest antibody response with intramuscular injection of Ad5. This lack of antibody response was consistent with previous studies looking at the response to a single Ad5 vaccination [55], [56]. Although the antibody levels were modest, 10^9^ and 10^10^ vp gave the highest levels of antibody.

Vaccinia-Gag viral challenge studies determined that two adjuvants, SP-D-4-1BBL and SP-D-BAFF, were the most promising in regards to protection from viral challenge. Similarly, in studies by the Watts lab, full length 4-1BBL enhanced responses to an Ad5 vaccine [34]. Their data also confirmed that 4-1BBL enhances antigen specific T cell responses ex vivo. In more recent studies, Moraes et al [35] observed enhanced protection of mice from lethal influenza challenge following vaccination with Ad5 expressing NP antigen combined with Ad5 expressing full length 4-1BBL. These data support our observation that 4-1BBL enhances the generation of memory CD8 T cells and enhances protection from viral challenge. Our data show that Ad5 viral vector vaccines expressing soluble multimeric 4-1BBL and HIV-1 Gag antigen can enhance the protection of mice from vaccinia-Gag viral challenge. Surprisingly, we did not observe a significant increase in dextramer-specific CD8 T cells with SP-D-4-1BBL, despite protection from vaccinia-Gag challenge. Increased numbers of dextramer positive cells were observed in prior studies [34], [35]. Despite this lack of a measurable difference in immune markers, SP-D-4-IBBL was an effective adjuvant, able to significantly reduce vaccinia-Gag viral titers (Fig. 4B) and induced a robust lymphocyte-mediated inflammatory response within the genital track of infected female mice (Fig. 5).

BAFF has not previously been tested as an adjuvant for Ad5 viral vector vaccines. Our data suggest that soluble multi-trimers of BAFF are able to enhance CD8+ T cell responses against vaccinia infection. We observed complete protection from vaccinia-Gag challenge in 3 of 5 mice. A significant increase in dextramer-specific CD8+ T cells and IFN-γ ELISPOT responses was also observed. Therefore BAFF is an intriguing adjuvant for T cell-mediated immunity. BAFF plays a central role in the development of B cell memory through the B cell receptor (BAFFR) [43]–[45]. BAFFR is also expressed on CD4+ T cells [57], [58] where it is able to co-stimulate T cell responses. Another possibility is that SP-D-BAFF is co-stimulating CD4+ T cells that then provide help for the CD8+T cell responses that provide protective immunity through a yet to be determined mechanism. The ability of BAFF to stimulate B cell responses suggests that SP-D-BAFF has the potential to enhance both T cell and antibody-mediated immunity. However, we did not observe increased antibody responses with Ad5-SP-D-BAFF. Nevertheless, there is evidence that BAFF can enhance antibody responses in DNA prime/protein boost vaccines [59], [60].

Mice vaccinated with the SP-D-BAFF and SP-D-4-1BBL adjuvants and then challenged with vaccinia-Gag displayed a significant reduction in virus load and were also the only groups that presented with inflammation in the ovaries and uterus (Fig 5). We propose that the large number of infiltrating lymphocytes we observed by histology are responsible for the reduction in viremia. In contrast, decreased levels of cytokines (IFN-γ, IL-12p70, MCP-1) by day 5 most likely represents the clearance of virus from the tissue (Fig. 5E). We did not observe a change in the levels of IL-6, TNF-α, and IL-10 between groups (data not shown). These data likely represent variation in these cytokines irrespective of the level of viremia. These data highlight a potentially unique mechanism of protection induced by multi-trimeric 4-1BBL and BAFF molecular adjuvants, perhaps via the generation of a unique T cell phenotype. For example, recent evidence suggests that 4-1BB stimulation generates T cells expressing high levels of Eomesodermin [61], [62] and these T cells are highly active for cytolytic activity. Our data suggest that SP-D-BAFF and SP-D-4-1BBL generate similarly enhanced cytolytic activity. Future studies will explore the mechanism of this response.

Surprisingly, IL-12p70 did not offer any protection to vaccinia challenge, despite its ability to induce a significant increase in dextramer-positive T cells (Fig. 7B). IL-12 has been evaluated in many studies as a DNA viral vector adjuvant [23]. IL-12 plays a central role in T-cell priming and proliferation during the interaction of T cells with antigen presenting cells. Despite the fact that we observed the highest levels of dextramer-specific cells with IL-12p70 adjuvant, these antigen specific CD8 T cells appear to offer little immunological benefit following vaccinia challenge. Presumably IL-12 is not capable of generating a high quality immune response in the manner of SP-D-4-1BBL and SP-D-BAFF. This difference could not be quantified by the standard immune assays we performed, suggesting that new assays are required to distinguish suboptimal T cells generated by IL-12p70 from highly effective anti-viral T cells generated by SP-D-BAFF and SP-D-4-1BBL. One possibility is that SP-D-BAFF and SP-D-4-1BBL generate a distinct form of T cell phenotype that can mount the dramatic inflammatory response we observed, while IL-12p70 induces a response that is limited in its ability to rapidly remove infected cells. This may be reflected in the smaller proportion of T_cm_ cells we observed with IL-12p70 adjuvant, but does not explain the robust T_cm_ response by SP-D-GITRL that failed to induce protective immunity following vaccinia-Gag challenge.

SP-D-4-1BBL and SP-D-BAFF adjuvants had a remarkable effect on lymphocyte trafficking into virus-infected tissues, where the ovaries and uterus of mice are known to be especially permissive for the replication of vaccinia (a form of cowpox) [63]. This has important implications for an HIV vaccine for which immune control is critically dependent upon strong cellular immune responses at the site of infection [64]. For example, the presence of CD8+ T cells in the genital mucosa was closely associated with control of infection in macaques infected with SIV by the intravaginal route [65]. From this and other studies, an emphasis has been placed on designing an HIV vaccine that delivers CD8+ T cells to mucosal sites in order to protect against sexual transmission [66], [67]. However, CD8+ T cells do not always traffic into tissues, which has been a special problem when CD8+ T cells are used for tumor immunotherapy [68]. While BAFF has not been studied in this context, it is interesting that 4-1BB stimulation has been shown to promote CD8+ T cell entry into the tumor microenvironment [69], [70]. While these studies used an agonistic anti-4-1BB antibody, the present report shows how a vaccine strategy using SP-D-4-1BBL can promote lymphocyte migration into diseased tissues. Further studies are needed to delineate the specific mechanisms involved in this process.

The evidence of a strong inflammatory immune response to viral challenge (edema and vasculitis in the genital tract) presents a number of potential benefits, but also challenges, for the design of an HIV vaccine. Rapid infiltration of T cells to HIV-1 infectious foci within the genital tract [71], [72] could rapidly clear the virus from the genital tract before spread of virus to the lymphoid organs [73]. In contrast, the inflammatory response may increase the number of activated CD4+ T cells and macrophages at the site of HIV-1 infection, leading to more rapid dissemination of the virus by “adding fuel to the fire” [74]. Studies of SP-D-BAFF and SP-D-4-1BBL in non-human primates would allow us to examine these questions. There are also issues related to the design of HIV-1 vaccines using Ad5 vectors. Recent clinical trials showed Ad5 viral vector HIV-1 vaccines did not enhance protection from HIV-1 acquisition, and there is evidence of increased HIV-1 infection rates in vaccinees with prior exposure to Ad5 [5], [75]. Whether 4-1BBL and BAFF adjuvants do overcome issues related to Ad5 vectors will need to be explored further. We are currently examining the expression of SP-D-TNFSF ligand adjuvants for other viral vectors vaccines, including MVA.

To date, a number of immune correlates of protection to viral challenge have been proposed. In this study, the only immune assay that correlated with reduced viremia was IFN-γ ELISPOT. The number of central memory T cells for each group suggested that SP-D-4-1BBL and SP-D-BAFF generated increased T_cm_ numbers, although a significant increase in the T_cm_ cell population was also observed for IL-12p70. Overall, these data suggest that future studies are required to determine the T cell phenotype that best correlates with enhanced protection from virus challenge.
